# Thymoquinone protects the testes of hypothyroid rats by suppressing pro-inflammatory cytokines and oxidative stress and promoting SIRT1 testicular expression

**DOI:** 10.3389/fphar.2022.1040857

**Published:** 2022-11-24

**Authors:** Sami A. Algaidi, Khadija A. Faddladdeen, Ghadeer I. Alrefaei, Safa H. Qahl, Emad A. Albadawi, Hailah M. ALmohaimeed, Nasra N. Ayuob

**Affiliations:** ^1^ Department of Anatomy, College of Medicine, Taibah University, Medina, Saudi Arabia; ^2^ Department of Biology, Faculty of Science, King Abdulaziz University, Jeddah, Saudi Arabia; ^3^ Department of Biology, College of Science, University of Jeddah, Jeddah, Saudi Arabia; ^4^ Department of Basic Science, College of Medicine, Princess Nourah Bint Abdulrahman University, Riyadh, Saudi Arabia; ^5^ Department of Medical Histology, Faculty of Medicine, Damietta University, Damietta, Egypt; ^6^ Yousef Abdullatif Jameel Chair of Prophetic Medical Applications (YAJCPMA), Faculty of Medicine, King Abdulaziz University, Jeddah, Saudi Arabia

**Keywords:** thymoquinone, testis, hypothyroidism, rat, SIRT1, TNF-α, NF-κB, PCNA

## Abstract

**Background:** Hypothyroidism has been linked to many testicular structural and dysfunctional changes in males. Thymoquinone (TQ) has shown a potent testicular protective effect through its antioxidant, anti-inflammatory, antiapoptotic, fertility-enhancing, and endocrine modulatory activities.

**Objectives:** This study aimed to investigate the efficacy of TQ in preserving the testicular structure of a model of experimentally induced hypothyroidism in rats and identify the mechanism behind this effect.

**Materials and methods:** Propylthiouracil (PTU) was used to induce hypothyroidism in adult male Wistar rats, who were then treated with TQ (50 mg/kg/body weight) for 4 weeks and compared to the untreated rats. Thyroid hormonal profile, oxidants/antioxidants profile, and serum testosterone levels were assessed. Gene expression and immune expression of SIRT1 and pro-inflammatory cytokines TNF-α and NF-κB were also assessed in the testicular tissue.

**Results:** TQ administration successfully improved PTU-induced disturbance in the thyroid hormonal profile (T3, T4, and TSH), serum testosterone level, and pancreatic antioxidants compared to the untreated hypothyroid group. TQ significantly downregulated (*p* = 0.001, *p* ˂ 0.001) TNF-α and NF-κB transcription, while it significantly upregulated (*p* = 0.01) SIRT1 transcription in the testes of hypothyroid rats. TQ markedly relieved the histopathological testicular changes induced by PTU and significantly increased (*p* = 0.002, *p* = 0.01) the sectional area of seminiferous tubules and germinal epithelial height, respectively. TUNEL-positive apoptotic germinal cells were significantly decreased (*p* ˂ 0.001), while PCNA-positive proliferating germinal cells and androgen receptor expression were significantly increased (*p* ˂ 0.001) in the testes of TQ-treated hypothyroid rats.

**Conclusion:** Thymoquinone could limit the hypothyroidism-induced structural changes in the testis, mostly through the upregulation of SIRT1 expression, which seems to mediate its promising antioxidant, anti-inflammatory and antiapoptotic effects that were evident in this study. Therefore, TQ is recommended as an adjuvant safe supplement in managing hypothyroidism, especially in males.

## Introduction

The testes are among the tissues that have abundant thyroid hormones receptors functioning throughout life and are concerned with regulation of cellular metabolism. Thyroid hormone (TH) has a crucial role in spermatogenesis and steroidogenesis and control of Sertoli cell proliferation and functional maturation, as well as in Leydig cell differentiation during the postnatal period (La Vignera and Vita, 2018; Hernandez and Martinez, 2020). The prevalence of overt thyroid disease is high. For example, approximately 10% of subjects tested for thyroid diseases have subclinical hypothyroidism (Vanderpump, 2019). Hypothyroidism in males has been linked to hypogonadism, reduced serum testosterone, and altered sperm morphology and motility (Krysiak et al., 2020).

Thyroid hormones (THs) play an essential physiological role in reducing the oxidative stress (OS) that is induced by reactive oxygen species (ROS). Therefore, the absence or deficiency of THs results in hypothyroid status and oxidative stress and is commonly resulted in testicular dysfunction and infertility (Naseem et al., 2019). Oxidative stress and inflammation are mitochondrial reactive species that are considered to be signaling molecules that mediate the production of pro-inflammatory cytokines. Therefore, OS is related to both systemic inflammation and hormonal derangement (Siti et al., 2015).

*Nigella sativa* L., which is also known as black seed, is one of the most popular, effective, and widely used medicinal herbs around the world (Ahmad et al., 2021). Previous studies on humans have demonstrated that *Nigella sativa* is safe, which has encouraged investigations of its efficacy in treating many diseases (Tavakkoli et al., 2017). For example, it has been reported to effectively protect the thyroid cell and reduced hypothyroidism-induced OS (Khalawi et al., 2013). Reduced TH level is described to be associated with reduction in the antioxidant defense with subsequent loss of testicular defense mechanism against ROS produced by the cellular metabolism (Sahoo et al., 2008). Thymoquinone (TQ), a prominent bioactive flavonoid of *Nigella sativa*, has a highly potent testicular protective effect *via* its antioxidant, anti-inflammatory, antiapoptotic, fertility-enhancing, and endocrine and immune-modulatory effects (Hassan et al., 2019; Wani et al., 2022).

SIRT1 is a nuclear protein that regulates critical metabolic and physiological processes in all of the body’s systems and specifically in the male reproductive system. It is mostly expressed in spermatocytes and spermatogonia and has been linked to male germ cell development (Conde et al., 2012; Bell et al., 2014). Both TH and SIRT1 are correlated and it has been reported that SIRT1 co-regulates the TH receptor-mediated induction of gene expression (Suh et al., 2013). In addition, TH may be a regulator of SIRT1 expression (Cordeiro et al., 2013). SIRT1 was described to have an important role in pathogenesis of chronic diseases (Thirupathi and De Souza, 2017). Therefore, SIRT1 expression was assessed, in the present study, as an indirect indicator of the spermatogenesis.

These observations represent a rational to hypothesize that supplementation with TQ can be an effective candidate against hypothyroid-induced testicular affection. However, there is a lack of the scientific evidence from the previous studies to explain this potential protective effect of TQ, which has motivated our study. Therefore, this study aims to test the efficacy of TQ in preserving the testicular structure in hypothyroid-induced rats and will explore the molecular bases of this effect.

## Materials and methods

### Chemicals

Thymoquinone was obtained from Frinton Laboratories, Inc. It was diluted with dimethyl sulfoxide (DMSO) (1:100) and given at a dose of 50 mg/kg/body weight/day through the gastric tube (Sankaranarayanan and Pari, 2011 #333).

Experimental hypothyroidism was induced in rats using propylthiouracil (PTU) (Sigma-Aldrich Inc. Hainesport, United States), which was given *via* gastric tube at a dose of 6 mg/kg/body weight daily for 6 weeks (Villar et al., 1998).

### Experimental animals

In total, 24 adult male Wistar rats weighing 180–200 g were obtained from King Fahed Medical Research Center (KFMRC). Acclimatization for 2 weeks under the standard laboratory conditions was allowed. Rats were fed the standard basal diet and water during the whole experiment (Table 1). The rats were randomly assigned into four groups (*n* = 6). Then, 1 mL of dimethyl sulfoxide (DMSO) in saline was administrated to the control group (NC) *via* gastric tube for 6 weeks. Thymoquinone was administrated to the positive control group (TQ) for 6 weeks. Hypothyroidism was induced in the other two groups *via* PTU administration and after 2 weeks of PTU administration, the hypothyroidism was assessed by measuring the triiodothyronine (T3), thyroxine (T4), and thyroid-stimulating hormone (TSH) levels in the serum. The rats were then further allocated into two groups: the hypothyroid group continued on PTU alone for another 4 weeks and the hypothyroid + TQ group was treated with TQ (while they were continued on PTU) for 4 weeks.

**TABLE 1:** Components of the basal diet of the rats.

Ingredients	Standard diet %
Casein	15
Corn starch	65
Sunflower oil	10
Fiber	5
Mineral mixture	4
Vitamin mixture (A, D, E, K, B1, B2….)	1
Total	100

### Sampling

The rats were euthanized at the end of the experiment by cervical dislocation after being anesthetized with 4% isoflurane (SEDICO Pharmaceuticals Company, Cairo, Egypt) in 100% oxygen. Blood samples were obtained by heart puncture, and were then centrifuged. The serum was kept at −80°C until it was used for the biochemical analysis. Levels of T3, T4, and TSH (Bayer HealthCare) were assessed in the serum using ADVIA Centaur automated competitive chemiluminescence immunoassay. The serum level of testosterone was estimated according to Zanoli et al. (2005).

### Assessment of stress markers

After cervical dislocation under anesthesia, the testes were rapidly dissected out. Parts of the left-hand testes were used for assessment of stress markers. They were homogenized and centrifuged for 10 min at 5000x g. The supernatant was utilized to assess the testicular levels of reduced glutathione (GSH), nitric oxide (NO) and malondialdehyde superoxide dismutase (SOD), catalase (CAT), and glutathione peroxidase (GPX) (Biodiagnostic, Giza, Egypt).

### Biochemical studies

The right-hand testes were fixed in 10% neutral buffered formalin for 24 h then processed into paraffin blocks. Parts of these paraffin-fixed testes underwent RNA extraction, as previously described (Pikor et al., 2011). TRIzol was used for total RNA extraction based on the supplier’s instruction (Invitrogen Life Technologies, Carlsbad, CA, United States). The Nano Drop 2000 Spectrophotometer (Thermo Scientific, United States) was utilized to estimate the concentration of RNA. Reverse transcription was done using oligo-dT primers (Bioneer Inc., Daejeon, Republic of Korea) in a 20-ll reaction including 5 ll RNA. The resulted cDNAs were amplified using PCR Master Mix (Bioneer Inc., Daejeon, Republic of Korea) with primers (Metabion International AG, Semmelweisstr, Germany), as was previously described (Pikor et al., 2011).

The results were analyzed using LightCycler 480 software (version 1.5, Roche Applied Science, Mannheim, Germany). The 2^-ΔΔCt^ method was used to estimate the relative levels of mRNA. For GAPDH (forward) CAA​CTC​CCT​CAA​GAT​TGT​CAG​CAA-3′, (backward) 5-'GGC​ATG​GAC​TGT​GGT​CAT​GA-3′, for tumor necrosis factor- α (TNF-α) 5′-CCC​TGG​TAC​TAA​CTC​CCA​GAA​A-3' (forward) and 5′-TGT​ATG​AGA​GGG​ACG​GAA​CC-3' (backward), for SIRT1 5′-GTG GCA GTA ACA GTG ACA GTG-3' (forward) and 5′-GTC AGC TCC AGA TCC TCC AG-3′ (backward), NF-κB, 5′-CGGATGACAGAGGCGTGTATT-3′(forward) and 5′- CGA​ACU​CAC​UGG​UCU​GAC​C -3′ (backward), and Sirtuin 1 (SIRT1) 5′-GTG GCA GTA ACA GTG ACA GTG-3' (forward) and 5′-GTC AGC TCC AGA TCC TCC AG -3' (backward).

### Histopathological examination and immunohistochemical study

The paraffin-processed blocks of the testes were sectioned at 4 μm and stained with hematoxylin and eosin for histopathological examination using a light microscope (Olympus, United States) that was connected to a digital camera. Some slides were stained immunohistochemically, as was described by Nie, Zhou, and Jassim (2002). The proliferating cells were visualized immunohistochemically using rabbit polyclonal primary antibody for anti-proliferating cell nuclear antigen (PCNA) (Santa Cruz Biotechnology, sc7907, at a dilution of 1:200). The rabbit anti-AR (Biocare Medical, Pacheco, AR441, United States, at a dilution of 1/100) primary antibody was used to localize the androgen receptor (AR). The anti-TNF-α (Abcam, ab6671, UK at the dilution of 1:500) primary antibody was used. The anti-nuclear factor kappa B (NF-κB; Dako, S2369, United States at a dilution of 1/100) primary antibody was utilized. The anti-SIRT1 (Santa Cruz Biotechnology, sc-74465, CA, United States at a dilution of 1: 200) primary antibody was used at a dilution of 1: 200. The primary antibody was omitted in some sections to be utilized as negative controls. All of the positive reactions were distinguished as principally nuclear and cytoplasmic by brown staining.

Regarding the *in situ* apoptosis detection, antigen retrieval (terminal deoxynucleotidyl transferase dUTP-mediated nick-end labeling, TUNEL assay) was performed using the TACS-XL DAB *in situ* apoptosis detection kit, Trevigen, Inc (Helgerman, United States) according to the manufacturer’s instructions. Paraffin sections, sectioned at 3 μ, from the right-hand testes were mounted on glass slides, pretreated with 3-amino propyltriethoysilane, and warmed at 37°C overnight and were then processed as was previously described (Rajeh et al., 2012).

An Olympus BX-51 microscope (Olympus) connected to a digital camera and a computer was used for photographing. Image-Pro Plus software version 6.0 (Rockville, United States) was used for semiquantitative analysis of antibody immunoreactivity. The area percentage of NF-κB, TNF-α, and SIRT1 expression was assessed in 30 fields using a ×40 objective lens and ×10 ocular lens, which was used as an indicator of the extension of the reaction. The number of TUNEL and PCNA-positive cells was counted. The cross-sectional area of the seminiferous tubules (STs) and the germinal epithelial thickness lining the STs were assessed in the testes in five non-overlapping sections in each animal at magnification ×100 using the same program. In total, 10 readings from each section were taken and the mean for each animal was calculated.

### Statistical assessment

In this study, the raw data were analyzed using the Statistical Package for the Social Sciences (SPSS, version 16; SPSS Inc., Chicago, Illinois, United States). The results are presented in the form of mean ± standard deviation. Analysis of variance ANOVA (f-test) was used to compare the study groups followed by Bonferroni *post hoc* test to avoid a multiple comparison effect. Significance was considered at *p*-value < 0.05.

## Results

### The effect of TQ on serum T3, T4, and TSH

PTU administration, in this study, has resulted in a significant reduction in T3 (*p* = 0.01) and T4 (*p* ˂ 0.001) levels, while it significantly increased the (*p* ˂ 0.001) TSH level when compared to the control group. T3, T4, and TSH levels did not show a significant change in the TQ-treated group when compared to the control. The hypothyroid group treated with TQ showed a significant increase in T3 (*p* = 0.04) and T4 (*p* = 0.004) levels, while it significantly reduced the (*p* ˂ 0.001) TSH level when compared to the untreated hypothyroid group (Table 2).

**TABLE 2:** Effect of thymoquinone on the serum testosterone level, thyroid hormonal profile, and oxidants/antioxidants profile.

Study variables	NC	TQ	Hypothyroid	Hypothyroid + TQ
Serum testosterone (ng/ml)	17.69 ± 1.65	16.60 ± 2.40 *p* = 0.76\1	5.89 ± 1.94 *p*˂0.001\1	13.02 ± 4.51 *p* = 0.06\1
				p1 = 0.002
T3 (ng/ml)\1	128.82 ± 11.15	130.90 ± 7.57 *p* = 0.86\1	105.80 ± 11.68 *p* = 0.01\1	125.64 ± 14.35 *p* = 0.76\1
				p1 = 0.04
T4 (ng/ml)\1	3.50 ± 0.82	3.48 ± 0.68	1.55 ± 0.73 *p*˂0.001\1	3.21 ± 0.63 *p* = 0.98\1
		*p* = 0.95		p1 = 0.004
TSH (mIU/L)\1	3.61 ± 1.18	3.53 ± 1.01 *p* = 0.65\1	19.53 ± 1.42 *p*˂0.001\1	2.91 ± 1.05 *p* = 0.67\1
				p1˂0.001
GPX (mU/mL)\1	156.00 ± 22.31	172.17 ± 21.29 *p* = 0.87\1	182.78 ± 21.13 *p* = 0.18\1	158.66 ± 13.74 *p* = 0.76\1
				p1 = 0.93
SOD (U/mL)\1	281.71 ± 18.63	306.79 ± 12.38 *p* = 0.06\1	105.61 ± 232.42 *p*˂0.001\1	232.42 ± 17.06 *p*˂0.001\1
				p1˂0.001
CAT (U/mL)\1	291.97 ± 17.91	312.00 ± 87.06	544.67 ± 13.47 *p*˂0.001\1	423.45 ± 81.35 *p* = 0.01\1
		*p* = 0.67		p1 = 0.02
MDA (µmol/L)\1	6.57 ± 1.30	3.75 ± 1.37	19.79 ± 1.93 *p*˂0.001\1	13.51 ± 2.90 *p*˂0.001\1
		*p* = 0.14		p1˂0.001
NO (µmol/L)\1	25.64 ± 2.66	26.18 ± 5.01 *p* = 0.67\1	48.04 ± 2.64 *p*˂0.001\1	33.49 ± 6.16 *p* = 0.03\1
				p1˂0.001
GSH (mmol/L)\1	0.43 ± 0.08	0.40 ± 0.12 *p* = 0.87\1	0.24 ± 0.06 *p* = 0.01\1	0.53 ± 0.09 *p* = 0.42\1
				p1˂0.001

One-way analysis of variance was used to compare the studied groups followed by Bonferroni post hoc test. Results are presented in the form of mean ± standard deviation (Diseases). Significance was considered when *p* < 0.05.

*p*, significance versus the control.

p1, significance versus the hypothyroid.

### The effect of TQ on the oxidant/antioxidant profile

The hypothyroid state that was induced by PTU in this study resulted in a disturbance in the oxidant/antioxidant profile, which presented with a significant upregulation (*p* ˂ 0.001) of MDA and NO levels when compared to the control group. Administration of TQ induced a significant reduction (*p* ˂ 0.001) in the level of MDA and NO in hypothyroid rats when compared to the untreated hypothyroid group. MDA and NO levels of TQ-treated hypothyroid rats showed a significant increase (*p* ˂ 0.001, *p* = 0.03) when compared to the control rats (Table 2).

When it came to the levels of antioxidants, it was observed that the testicular levels of SOD, CAT, and GSH were significantly reduced (*p* ˂ 0.001, *p* ˂ 0.001, *p* = 0.01) in hypothyroid rats when compared to the control. Although there was a significant upregulation (*p* ˂ 0.001, *p* = 0.02, *p* ˂ 0.001) in SOD, CAT, and GSH of TQ-treated hypothyroid rats when compared to the untreated rats, respectively, the levels of SOD and CAT were still significantly high (*p* ˂ 0.001, *p* = 0.01) when compared to the control. GPX levels did not show any significant changes across the studied groups (Table 2).

### The effect of TQ on the serum level of testosterone

Upon assessing the effect of hypothyroidism on the testosterone level, it was noticed to be significantly reduced (*p* ˂ 0.001) in hypothyroid rats when compared to the control group, while TQ administration to hypothyroid rats significant increased (*p* = 0.002) when compared to the untreated rats. The control rats that received TQ did not show any significant change in testosterone levels when compared to the control rats (Table 2).

### The effect of TQ on the gene expression of TNF-α, NF-κB, and SIRT1

**FIGURE 1:**
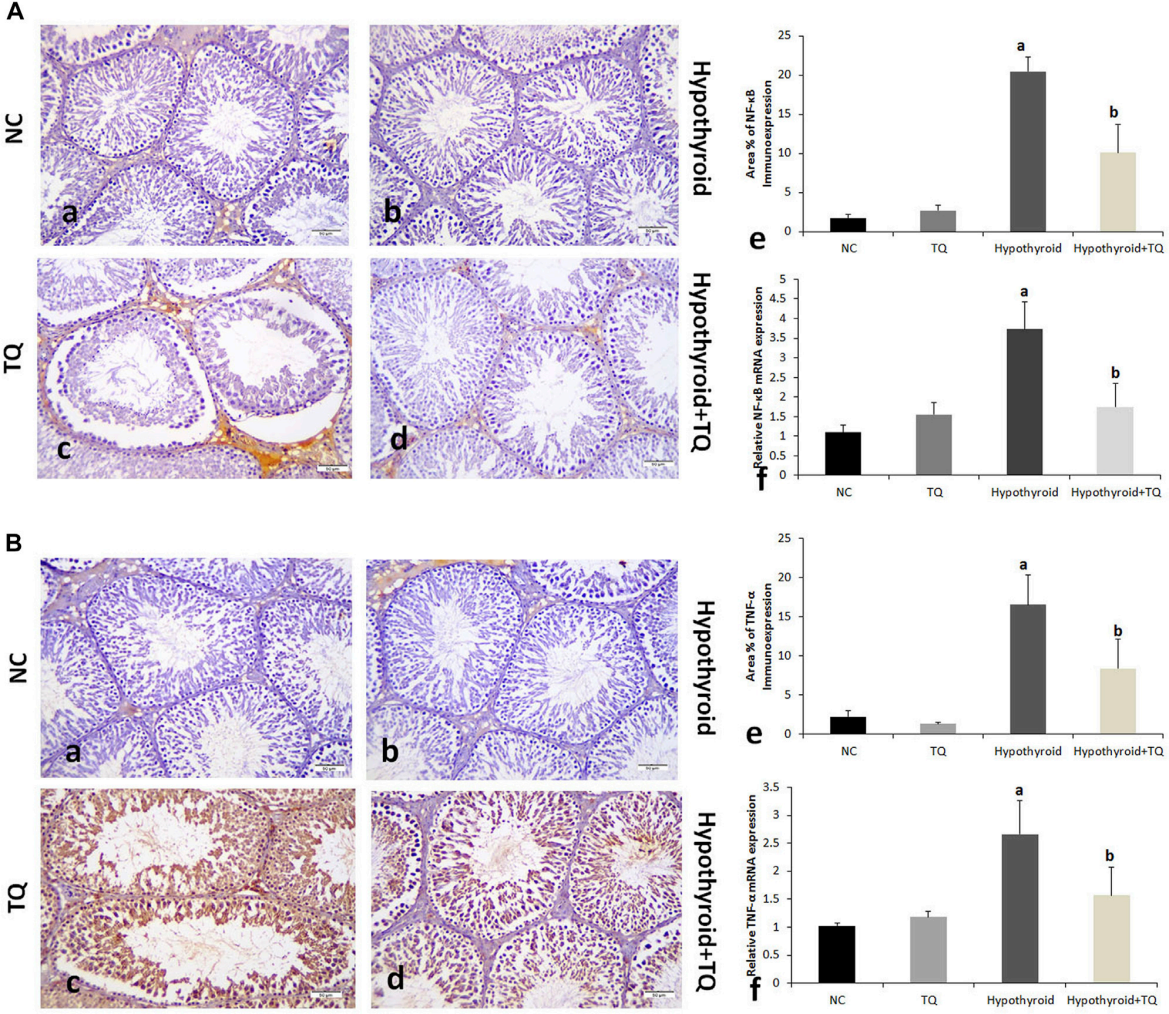
Effects of thymoquinone administration on the histological structure of the testes of hypothyroid rats **(A)**. Control (a) and TQ-treated (b) groups show intact structure, while the hypothyroid group (c) shows smaller and irregular ST, widening of the interstitial space, and reduced number and vaculation of interstitial cells of Leydig (black arrow). The germinal epithelium showed multiple spaces and gaps between the cells, detachment, and reduced spermatocytes density (red arrow). The ST showed reduced number and scattered spermatids (star). These changes are less frequently observed in the hypothroid + TQ group (d). The graph shows the cross-sectional area of the seminiferous tubules **(B)** and the height of the germinal epithelium **(B)** of the studied groups. TQ, Thymoquinone. NC, Normal control. Data are presented as the mean ± SD. A comparison between groups was done using one-way ANOVA test followed by Bonforoni post hoc test (*n* = 6).

The level of testicular TNF-α and NF-κB transcription was significantly upregulated (*p* ˂ 0.001) in the hypothyroid rats, while it showed a significant downregulation (*p* = 0.001, *p* ˂ 0.001) in TQ-hypothyroid rats when compared to the untreated hypothyroid rats (Figure 1).

**FIGURE 2:**
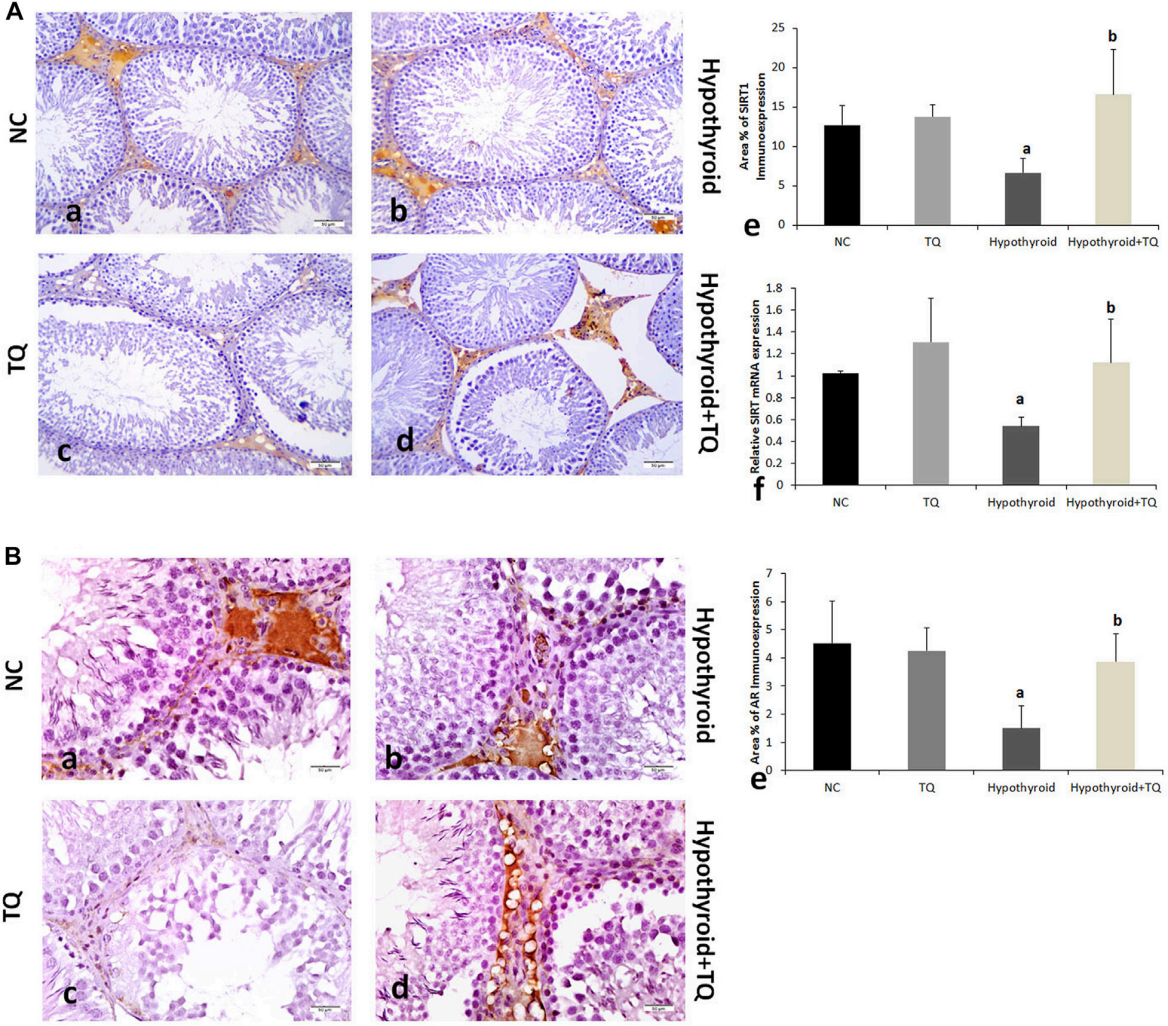
Effects of thymoquinone (TQ) administration on the immunoexpression of PCNA **(A)** and TUNEL **(B)**. Graphs that show the quantitative assessment of the number of PCNA-positive **(C)** and TUNEL-positive cells **(D)** are presented. TQ, thymoquinone. NC, normal control. Data are presented as the mean ± SD. Comparison between groups was carried out using one-way ANOVA test followed by Bonferroni post hoc test (*n* = 6).

For the effect of hypothyroid status on SIRT1 expression in testes, it was found that the transcription was significantly downregulated (*p* ˂ 0.001) in the hypothyroid rats, while it was significantly upregulated (*p* = 0.01) in hypothyroid rats treated with TQ when compared to the untreated hypothyroid rats (Figure 2). TQ administration to the control rats has no significant effect on TNF-α, NF-κB, or SIRT1 transcription in the testes (Figures 1, 2).

### The effect of TQ on histological findings

**FIGURE 3:**
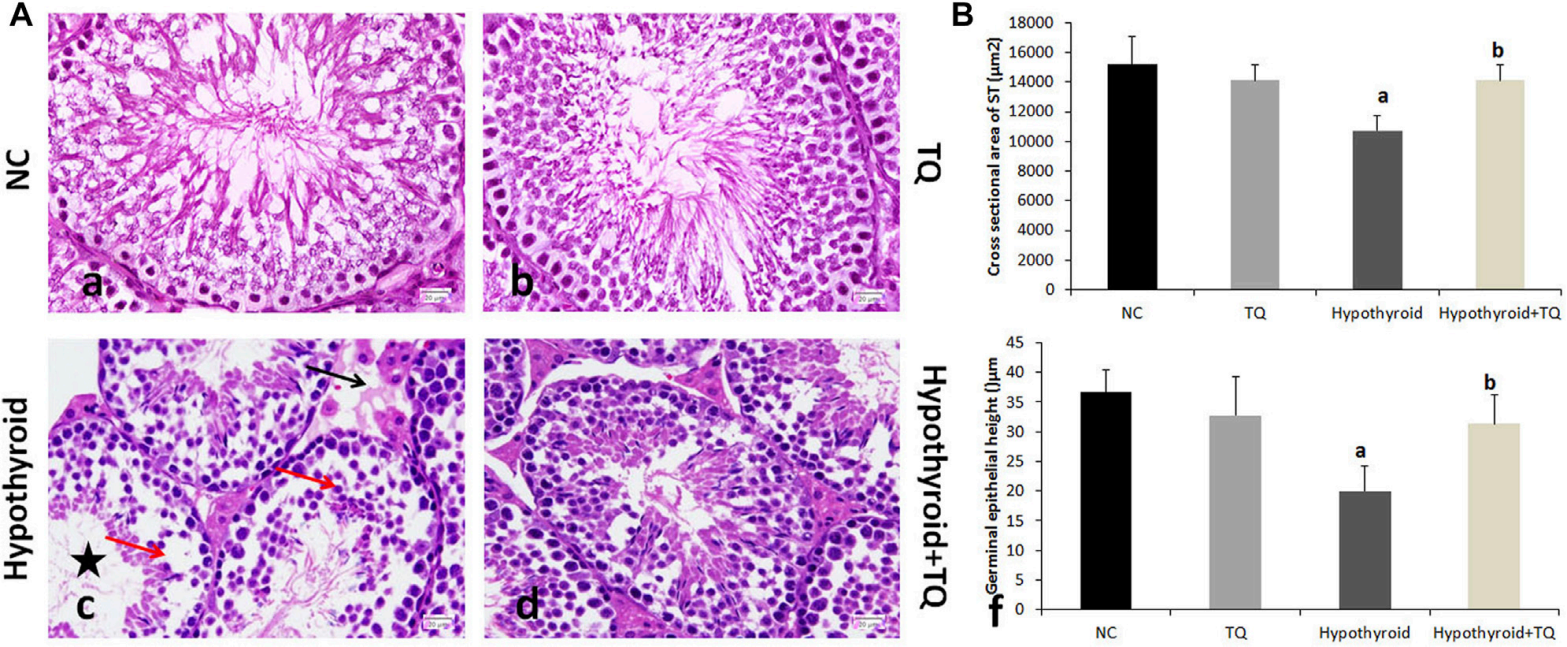
Effects of thymoquinone (TQ) administration on the expression of NF-κB **(A)** and TNF-α **(B)**. Representative pictures of immunoexpression of each marker are presented (a–d), in addition to graphs that show the quantitative assessment of immunoexpression (e) using Image-Pro Plus software version 6.0 and gene expression (f) assessed using RT-PCR. TQ, thymoquinone. NC, normal control. Data are presented as the mean ± SD. A comparison between groups was made using one-way ANOVA test followed by Bonferroni post hoc test (*n* = 6).

Both normal control and TQ-treated groups showed intact testicular structure in this study. However, PTU administration resulted in abnormal testicular histological changes, which included small sized and irregular shape STs, wide interstitial space, vacuolation, and a reduced number of interstitial cells of Leydig. The germinal epithelium showed multiple spaces between the cells, as well as detachment and reduced spermatocytes density. The lumen of STs showed few scattered spermatids. These changes are less frequently observed in the TQ-treated hypothyroid group (Figure 3).

A significant reduction (*p* ˂ 0.001) in the cross-sectional area of STs and germinal epithelial height was observed in the hypothyroid rats when compared to the control, while they were significantly increased (*p* = 0.002, *p* = 0.01) in hypothyroid rats treated with TQ when compared to the untreated hypothyroid rats (Figure 3).

**FIGURE 4:**
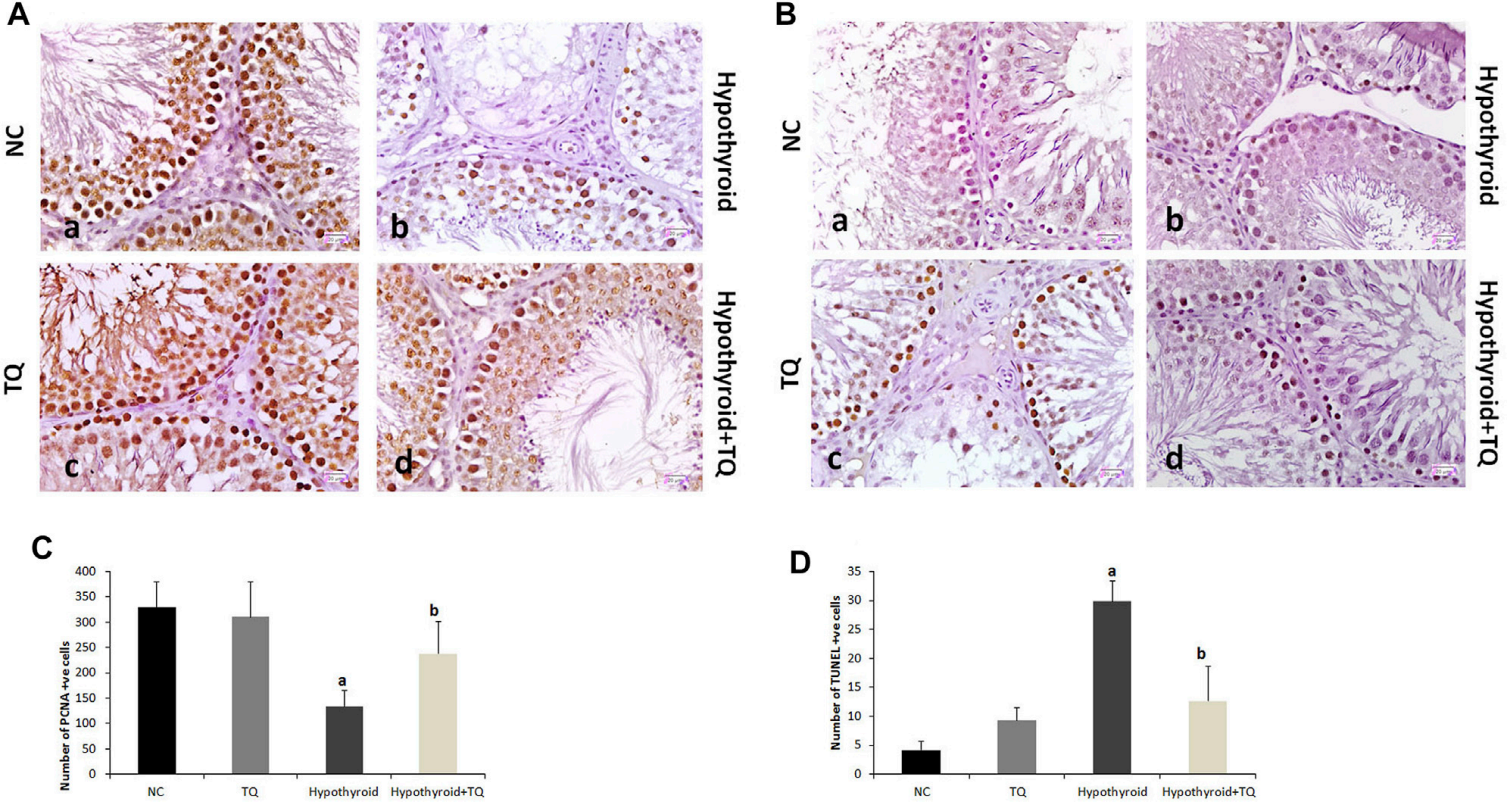
Effects of thymoquinone (TQ) administration on the expression of SIRT **(A)** and AR **(B)**. Representative pictures of immunoexpression of each marker are presented (a–d), in addition to graphs that show the quantitative assessment of immunoexpression. (e) using Image-Pro Plus software version 6.0 and gene expression (f) assessed using RT-PCR. TQ: thymoquinone. NC, normal control. AR, androgen receptors. Data are presented as the mean ± SD. A comparison between groups was made using one-way ANOVA test followed by Bonferroni post hoc test (*n* = 6).

Upon assessment of the effect of TQ administration on apoptosis in the testes of hypothyroid rats, the number of TUNEL-positive cells showed a significant increase (*p* ˂ 0.001) in hypothyroid rats when compared to the control, while it showed a significant reduction (*p* ˂ 0.001) in hypothyroid rats treated with TQ when compared to the untreated hypothyroid rats (Figure 4).

The germinal cells proliferation, which was indicated by the number of PCNA-positive cells, was significantly downregulated (*p* ˂ 0.001) in the testes of hypothyroid rats, while it showed a significant upregulation (*p* = 0.01) in TQ-treated hypothyroid rats when compared to the untreated rats (Figure 4).

Assessment of the inflammatory cytokines immunoexpression in the testes revealed that NF-κB and TNF-α were significantly upregulated (*p* ˂ 0.001) in hypothyroid rats when compared to the control, while they showed a significant downregulation (*p* ˂ 0.001) in TQ-treated hypothyroid rats when compared to the untreated rats (Figure 1).

Testicular SIRT1 immunoexpression was significantly reduced (*p* = 0.03) in hypothyroid rats when compared to the control, while it showed a significant upregulation (*p* ˂ 0.001) in TQ-treated hypothyroid rats when compared to the untreated rats (Figure 2). It was noticed that immunoexpression of AR was significantly downregulated (*p* = 0.03) in the testes of hypothyroid rats when compared to the control, while it showed a significant upregulation (*p* ˂ 0.001) in TQ-treated hypothyroid rats compared to the untreated rats (Figure 2).

## Discussion

The interrelationships between thyroid derangement, inflammation, and oxidative stress have previously been described. Inflammation leads to oxidative stress and affects thyroid function through hormone and cytokine changes. Hypothyroidism promotes the oxidative stress, which deteriorates hypothyroidism by inhibiting deiodinases and establish a vicious circle (Mancini and Di Segni, 2016). Therefore, this study aimed to test the efficacy of TQ as a potent antioxidant and anti-inflammatory substance in protecting the testes against hypothyroid-induced testicular changes in rats and explore the molecular bases of this effect.

In this study, an experimental induction of hypothyroidism using PTU was biochemically confirmed by the significant reduction in T4 and elevation of TSH levels, which is consistent with previous studies (Khalawi et al., 2013). A significant decrease in the antioxidants; SOD, CAT, and GSH, as well as a significant increase in MDA and NO in the testes of hypothyroid rats was also observed in this study. This is in line with the findings of Sahoo et al. (2008), who reported that hypothyroidism is associated with a reduction in antioxidant defense mechanism against free radicles produced during the normal cellular metabolism, with a subsequent accumulation of these free radicles, which resulted in abnormal function of the cells and increased cell death. The accumulated NO was reported to promote cell damage and led to inflammation and production of other free radicals from macrophages, Sertoli cells, and Leydig cells (Coştur et al., 2012; Bell et al., 2014). Meanwhile, TQ was reported to reduce oxidative stress, act as a radical scavenger, and thereby preserve the enzymatic activity of antioxidants such as catalase. This supports the observations in this study regarding the antioxidant activity of TQ.

In this study, the inflammatory status in the testes of hypothyroid rats was evident by the significant upregulation of the pro-inflammatory cytokines TNF-α and NF-κB at the genetic and cellular levels. NF-κB is considered to be a critical link between oxidative stress, inflammation, and apoptosis. Upon activation by oxidative stress, NF-κB up-regulates the inducible nitric oxide synthase (iNOS) level and leads to an increase in NO production, which subsequently triggers NF-κB upregulation. The latter initiates an inflammatory signaling cascade that triggers the release of numerous inflammatory cytokines (Aprioku, 2013). In this study, the testicular expression of TNF-α and NF-κB was significantly downregulated in TQ-treated hypothyroid rats, which indicates the anti-inflammatory actions of TQ. The latter was reported to be caused by the suppression of interleukin-1 receptor-associated kinase 1(IRAK)-linked activator protein (AP)-1/NF-κB pathways (Hossen et al., 2017).

Testicular expression of SIRT1 in hypothyroid rats was significantly downregulated in this study. This finding is supported by some previous studies, which reported that the proteins that protect against oxidative stress, such as SIRT1 and SIRT3 levels, were lower in peripheral blood samples from patients suffering from hypothyroidism than in healthy individuals (Al-Khaldi and Sultan, 2019). Reduced SIRT1 may be behind the negative impact of hypothyroidism on the testicular structure that was observed in this study. It was reported that SIRT1 deficiency is associated with attenuated spermatogenesis, germ cell function, reduced sperm counts, increased DNA damage, and apoptosis of spermatocytes (Coussens et al., 2008; Bell et al., 2014). In the current study, TQ administration resulted in upregulation of SIRT1 expression and downregulation of NF-κB in the testes of hypothyroid rats. This finding aligns with the work of Harphoush et al. (2019), who reported that TQ increased SIRT1 mRNA expressions (which is one of the genes that is involved in mitochondrial function and metabolism) in mammary gland and ovarian tissue of high fat diet-induced obese mice. They added that TQ induced its anti-inflammatory and antioxidant effects and improved the reproductive ability and obesity-associated metabolic dysfunction through the activation of genes involved in the AMPK/PGC1α/SIRT1pathway (Harphoush et al., 2019). Thirupathi and De Souza (2017) reported that SIRT1 plays an important role in the regulation of the pathogenesis of chronic diseases through its ability to deacetylate histones and non-histone proteins, such as NF-κB, as well as its ability to inhibit ROS production.

In this study, a significant decline in serum testosterone level was observed in hypothyroid rats, which is in line with the findings of Krytskyy and Pasyechko (2019). Decreased testosterone level was correlated to the reduced sex hormone-binding globulin (SHBG) synthesis in the liver, which was reported by La Vignera et al. (2017) in the hypothyroid state. The transfer of testosterone from the testes to brain tissue, where it is dissociated and crosses the blood–brain barrier to induce negative feedback on LH biosynthesis and secretion, occurs through SHBG. It has been postulated that hypothyroidism can induce a disruption in the thiol redox status of the mitochondria, which resulted in testicular structural and functional abnormalities (Ibrahim et al., 2021). Reduced testosterone level and AR expression might be also attributed to Leydig cell damage caused by the hypothyroid state-associated oxidative stress, which were evident both histopathologically and biochemically in this study. This explanation was recently reported by Krysiak et al. (2020), who mentioned that AR expression in Sertoli cells was reduced due to the state of oxidative stress. In addition, TQ administration significantly increased serum testosterone level and AR expression in the testis. This may be attributed to the preservation of Leydig cells through its antioxidant effect or its ability to enhance aromatase gene expression, which subsequently enhanced spermatogenesis (Aithal et al., 2016; Negi et al., 2018). In the same line of these findings, TQ has been described to have fertility-enhancing effect (Wani et al., 2022).

PTU administration in this study resulted in evident histological testicular changes, which included small irregular STs with few spermatids, degenerated Leydig cells, and reduced spermatocytes density. These findings are in agreement with those reported in different models of experimental hypothyroidism (Fadlalla et al., 2017; Hegazy et al., 2018; Ibrahim et al., 2021), which attributed these changes to apoptosis of these cells. Apoptosis of germ cells in the testes, which was observed in this study using the TUNEL technique, might be directly attributed to accumulated free radicals or indirectly to increased NF-κB. These histological changes were less frequently observed in the testes of TQ-treated hypothyroid rats. TQ was reported to markedly improve the structure of the testis and the quality of the sperm, due to its ability to downregulate Bax and caspase-3 apoptotic pathways (Hassan et al., 2019). In addition, the antioxidant defense of TQ against the generation of ROS might explain its efficacy in protecting the testicular tissue (Negi et al., 2018). Recently, TQ was also reported to block the release of apoptotic mediators and inflammatory cytokines, as was evident in our study, and subsequently inhibit the inflammatory process and apoptosis in the testicular tissue (Wani et al., 2022).

It has been reported that PCNA, which has a vital role in nucleic acid metabolism, is essential for DNA replication and repair in proliferating eukaryotic cells, and its expression regresses as the cells become inactive (Miura et al., 2002). In this study, PCNA-positive cells in the testes of hypothyroid rats was markedly reduced, which indicates DNA insults and damage of the actively dividing portion of the germinal epithelium with subsequent loss of the ability to repair. This is in accordance with the findings of Fadlalla et al. (2017). In this study, TQ administration induced a significant increase in PCNA-positive cells in the testes of hypothyroid rats, which indicates an improvement in the replicative ability of the germinal epithelium. Furthermore, the positive expression of PCNA is considered to be an indicator of testicular well-being (Adana et al., 2022).

Among the limitations of this study is the need for a more detailed understanding of the molecular mechanism of action of TQ on all parts of the male reproductive system in hypothyroidism.

In conclusion, thymoquinone was found to limit the hypothyroidism-induced structural changes in the testes, mostly through the upregulation of SIRT1 expression. This mediated its promising biological activities, which include the antioxidant and anti-inflammatory antiapoptotic effects that are evident in this study. Therefore, TQ is recommended as an adjuvant supplement in managing hypothyroidism, especially in males. Although further studies are required to provide a better understanding of the mode of action of TQ on the testes in hypothyroidism, we believe that the results of this study can easily be translated into clinical use given that TQ is a safe and natural product.

## Data Availability

The original contributions presented in the study are included in the article/Supplementary Material; further inquiries can be directed to the corresponding author.
